# Accelerating Translational Research through Open Science: The Neuro Experiment

**DOI:** 10.1371/journal.pbio.2001259

**Published:** 2016-12-07

**Authors:** E. Richard Gold

**Affiliations:** 1 McGill Faculty of Law, McGill University, Montreal, Quebec, Canada; 2 Department of Human Genetics, McGill University, Montreal, Quebec, Canada

## Abstract

Translational research is often afflicted by a fundamental problem: a limited understanding of disease mechanisms prevents effective targeting of new treatments. Seeking to accelerate research advances and reimagine its role in the community, the Montreal Neurological Institute (Neuro) announced in the spring of 2016 that it is launching a five-year experiment during which it will adopt Open Science—open data, open materials, and no patenting—across the institution. The experiment seeks to examine two hypotheses. The first is whether the Neuro’s Open Science initiative will attract new private partners. The second hypothesis is that the Neuro’s institution-based approach will draw companies to the Montreal region, where the Neuro is based, leading to the creation of a local knowledge hub. This article explores why these hypotheses are likely to be true and describes the Neuro’s approach to exploring them.

## Introduction

Translational research is often afflicted by a fundamental problem: a limited understanding of disease mechanisms prevents effective targeting of new treatments [1]. The linear commercialization model, led by large pharmaceutical companies and sponsored research projects [2], is an increasingly outdated and ineffective approach to remedying the problem [3–5]. Instead, biomedical stakeholders are implementing dispersed, network-based approaches to innovation [6,7] and emulating effective models from other high-tech industries [8,9]. Nowhere is this of more importance than in the field of neurodegenerative and neuropsychiatric disease [1].

Various “open” research models (re-)emerged over the past several decades to help accelerate research progress, with varying degrees of success [7,10]. Open Science at an institutional level is one such model. It represents both a “nuanced approach to dissemination of university knowledge” [4] sought by Nicol et al. and an evolution of the role of universities in the innovation system [11]. Seeking to accelerate research advances and reimagine its role in the community, the Montreal Neurological Institute (Neuro) announced in the spring of 2016 that it is launching a five-year experiment during which it will adopt Open Science across the institution, including all of its labs. This article explores the potential of Open Science in general, and at the Neuro and in Montreal in particular. Given the experimental nature of the initiative, the Neuro is funding projects to independently measure, compare, and assess its own performance and that of its Open Science initiative. While the Neuro’s story is unique in that it is the first institution to adopt an open science model across the entire spectrum of its research, which includes clinical work, it will also provide a window into the future applications of Open Science more generally.

In adopting Open Science at the institutional level, the Neuro hopes to achieve benefits beyond that for research; the initiative provides a foundation for multiple parties—researchers in and outside of McGill, patient organizations, regulators, and industry—to engage in neurobiological research and to engage in local “knowledge-based economic development” [11]. These benefits are expected to extend beyond the institution itself into the wider community. Open Science provides advantages over proprietary models of innovation in this respect by enhancing partnerships, lowering transaction costs, and encouraging local innovation where the subject matter of the innovation contains significant degrees of “sticky” (knowledge that is less expensive to use in place than to move elsewhere) [12], including tacit, knowledge.

## Definition

The term Open Science is the culmination of the last decade’s embrace of “open innovation,” “open access,” and “open data” [7]. By adding the eschewal of patent protection in addition to the features of these models, it better accords with the general public’s conception of the word “open.” The Open Science model aims at accelerating discovery, innovation, and research by encouraging rapid multilateral sharing of data, ideas, and materials without the limitations imposed by patent protection. Institutions adopting an Open Science platform seek to break down barriers between researchers, datasets, and partners to create dynamic knowledge hubs and eliminate artificial bottlenecks imposed on upstream research. The Neuro expects that partners will include researchers around the globe, other research institutions, commercial entities, and patients themselves. The Open Science model allows the “ingredients for successful innovation—skilled individuals, resources, and financing—to come together.” [5]

The Neuro’s Open Science model rests on principles that encompass a pledge to not seek patent rights over any of its research outputs at the institutional level—it currently applies for approximately five patents per year—while respecting each individual researcher’s independence to do so at her own expense. The model also promises open sharing of results, with the exception of clinical data supporting a regulatory application in line with the US Institute of Medicine’s recommendation [13]. Researchers will also be able to access associated metadata, physical biosamples, including through a soon-to-be established Neuro biobank, and other scientific materials. Drawing on tools already used by the McConnell Brain Imaging Centre, which currently has over 30,000 registered users worldwide and that is involved in international collaborations such as BigBrain (https://www.mcgill.ca/bic/home), the Neuro will develop a sharing infrastructure. The principles underlying the Open Science initiative recognize, however, that patient privacy and consent may, in some circumstances, limit what the Neuro shares. Finally, the Neuro expects its partners to uphold the same open principles in relation to the work they do directly with the Neuro (as opposed to their own follow-on or concurrent research).

The Neuro possesses unique characteristics that provide a particularly rich environment in which to test the benefits and weigh the costs of Open Science at the institutional level. These include housing clinicians, basic research, and sophisticated brain imaging facilities in a single institution. This improves the Neuro’s ability to link individual patient data, samples, and cells with clinical research and high-tech tools such as brain imaging databases without compromising researchers’ independence in setting research programs and pursuing discoveries. As the Open Science initiative takes form, the research group will explicitly and transparently collect data about its concrete effects on research, collaboration, model uptake by other institutions, and the local economy [8,11].

## Enhancing Partnerships

The Neuro’s initiative provides universities, policy-makers, and firms with the opportunity to evaluate whether Open Science enhances research and local economic growth. Specifically, it will allow these communities to examine two hypotheses.

The first of these hypotheses is that the Open Science initiative will attract new private partners. In fact, it has already done so. A number of these collaborated on a recent CDN$84 million grant from the Canadian federal government, and the Neuro is engaged in negotiations over significant partnerships (that will be announced when complete). In line with the Neuro itself, these partners are seeking solutions to struggling drug development and social responses to mental health issues. For example, because of the Open Science initiative, Thermo Fisher Scientific agreed to partner with the Neuro to develop reagents, including antibodies and knock-out cell lines, to accelerate research into a number of neurodegenerative diseases. Potential partners recognize the promise of the Open Science model, in particular the commercial benefits of sharing the risk of early-stage research, and accelerating the speed of research progress [6,8,14,15].

Similar forces launched the Structural Genomics Consortium (SGC) and the Allen Institute for Brain Science [9]. The Allen Institute is particularly pertinent, as it also focuses on neuroscience research and successfully implemented an open science policy almost 15 years ago. Its work has led to the creation of brain atlases, among other research tools, all of which are openly available, and some of which serve as standards in the field. The Neuro aims to develop this approach further by eliminating patents, moving into clinical work and associated data and by avoiding organizational challenges by preserving research independence. As the experience with the SGC illustrates, pharmaceutical companies have, in particular, expressed interest in participating in open science projects [5].

## Allowing for Knowledge Spillover

A second hypothesis is that the Neuro’s institution-based approach will draw companies to the Montreal region, where the Neuro is based, leading to the creation of a local knowledge hub with attendant jobs and attracting other firms with complementary specialties [16]. The Ubisoft-anchored video game cluster in Montreal illustrates this effect [16].

One of the expectations of the Neuro’s Open Science model is that it will lead firms to develop complementary downstream applications, creating advantages for translational research and developing a local knowledge hub. Because these applications will be complementary to the core research program, permitting partner firms to seek intellectual property protection does not sacrifice, or impede, scientific norms of openness. Open Science at the Neuro will allow these partners to pursue the legal avenues they choose on these complementary or downstream innovations. Further, Open Science also stimulates new research avenues: Murray et al. found that interaction with new partners uninhibited by restrictive intellectual property protection “encourag[es] the establishment of entirely new research directions,” and “reduce[s] the fixed cost of ‘entering’ a particular research area to conduct these investigations,” [17] while avoiding stigmatization of interesting avenues for follow-on research. “Faculty consulting” with private companies outside of the university’s purview causes a similar effect [11].

More generally, scholars have found that encouraging crossdisciplinary integration of expertise is a crucial component of overcoming roadblocks in research [11]. By maximizing ease of access and attracting new collaborators, many of whom are not specialized in the neurobiology domain, the Neuro will create an “interdisciplinary communit[y] made up of a heterogeneous range of members,” expected to accelerate the progression of neuroscience research and the growth of a “knowledge society” [14]. The Neuro’s aim is to build new algorithms, apps, and innovative software that will stimulate more activity, attract more firms and partners, and create a snowball effect. Future collaborations may form across a range of fields from finance to physics, to visualization software. Furthermore, the exchange of knowledge between specialists in different fields should help to avoid replication issues down the road and software bugs by diversifying the number of people with different backgrounds who look at given results.

## Lowering Transaction Costs

A further expectation worth testing is that Open Science lowers transaction costs, such as contract negotiations, court challenges, and intellectual property management that collectively impose a serious burden on universities [4]. Not only should decreasing these transaction costs attract new (particularly smaller) [9] partners by virtue of the increased simplicity of forming partnerships, but doing so can also be expected to accelerate research progress. More efficient resource allocation and decreasing management costs of institutions that specialize in basic research—not legal and business strategies—ought to further decrease costs [11]. Taking advantage of this cost reduction, the Neuro will be in a position to engage in open collaborations with firms, research consortia, and institutions around the world. Not being limited or constrained by the Neuro’s patents, new partner firms can develop business opportunities faster and at a lower cost than if the Neuro maintained a proprietary approach to its research.

Nevertheless, to fully access the benefits of Open Science, institutions need to invest in technicians, interoperability standards, and new infrastructure [8]. One would expect the cost savings from eliminating both patent protection and extended negotiations with partners to more than offset these costs [12]. Eliminating patent protections over life sciences research tools in particular will avoid raising the cost of “exploratory research that may enable the future creation of many applications, including those that still are undreamt of.” [14] Resources that are thus freed up can be reallocated to support research and innovation instead.

## Encourage and Intensify the Accumulation of “Sticky,” Including Tacit, Knowledge

A final advantage of the Neuro Open Science initiative is the expectation that it will accelerate the generation of sticky knowledge in the Montreal area. Knowledge is produced by universities but this is not often efficiently translated into local economic benefit. While spin-off companies provide one means through which to translate university-generated knowledge to the local economy [11], they are not the only means of doing so.

The Neuro’s knowledge hub—tools and approaches to analyzing the combination of genetic, brain imaging, and behavioral data, links between big data and individual patients, cell lines, and clinical expertise—creates knowledge that will be deeply embedded in Montreal and its surrounding region. This knowledge is not only key to identifying more promising targets for drug development and community mental health care [8] but is also sticky to Montreal as it is the result of positive externalities of innovation. It resides as tacit knowledge in the minds [14] and interaction of individuals living and working in Montreal [11]. Only by locating some operations in Montreal can firms fully take advantage of the ideas founded on the Neuro’s Open Science initiative [16].

## Conclusion

By encouraging new partnerships, reducing transaction costs burdening upstream research, and creating sticky knowledge specific to Montreal, the Neuro Open Science initiative is designed to promote local innovation development and dissemination of university knowledge [11]. The unique features of this initiative, namely the elimination of patent protection and its clinical institution-based nature, make it particularly well-suited to achieving these goals. Thanks to the Neuro’s commitment to independent and transparent monitoring, we will learn whether these hypothesized benefits turn into reality.

The movement away from traditional research models has begun, using other project-based “open” initiatives, and the Neuro Open Science initiative provides an important new piece in the puzzle of improving the efficacy of translational research. By itself, it may be able to accelerate some research. But to fully achieve the benefits of Open Science, other institutions will need to not only follow, but expand on, the Neuro’s lead.
